# Editorial: Device/pharmaceutical supported neuro-functional regeneration/recovery in neurological disorders

**DOI:** 10.3389/fneur.2024.1552118

**Published:** 2025-01-10

**Authors:** Eiichi Tsuda

**Affiliations:** Department of Rehabilitation Medicine, Hirosaki University Graduate School of Medicine, Hirosaki, Japan

**Keywords:** robotic therapy, wearable cyborg, brain stimulation, electrical stimulation, neurorehabilitation

The field of neuro-functional regeneration and recovery in neurological disorders has witnessed a remarkable evolution, driven by innovative devices and pharmaceuticals. This advancement is crucial given the profound impact neurological conditions, such as stroke, cerebral palsy, autism spectrum disorder (ASD), and developmental dyslexia, have on patients' quality of life. Recent research highlights the potential and limitations of various neuro-rehabilitation strategies, shedding light on their efficacy and the need for nuanced application tailored to individual patient profiles.

## Robotic therapy with wearable cyborg in stroke rehabilitation

The integration of robotics in neurorehabilitation, particularly for stroke patients, represents a significant leap forward. However, as the study by Wall et al. on electromechanically-assisted gait training with the wearable cyborg “Hybrid Assistive Limb (HAL)” revealed, this technology's benefits might not be as straightforward as hoped. The study, which compared conventional gait training to HAL-assisted training in stroke patients, found no significant differences in gait pattern function between the two groups. This prompts the researchers to challenge critical questions about how to optimize rehabilitation programs, particularly for those with moderate to severe gait impairments, and suggests that the success of robotic assistance may depend heavily on patient-specific factors and the timing of intervention.

## Expanding cyborg technology to pediatric care

**Figure 1:**
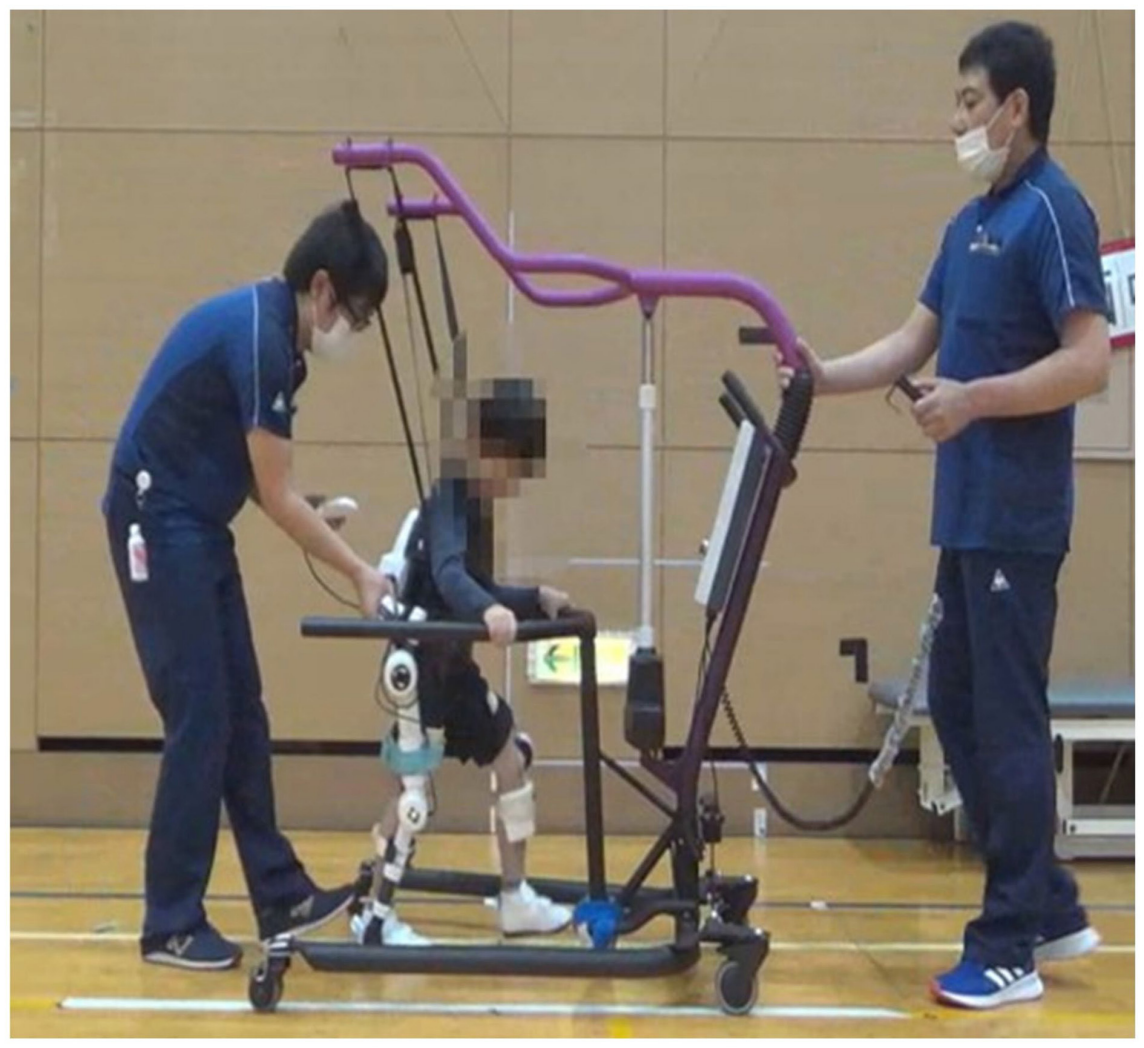
Neurorehabilitation using Hybrid Assistive Limb (HAL) of 2S size for a pediatric patient.

The exploration by Takahashi et al. on the newly-developed smaller HAL system (2S size) adapted for pediatric patients introduced a promising new frontier in treating motor function disorders in children with cerebral palsy and spinal cord disorders (Figure 1). The study findings, indicating improved motor function and patient satisfaction without serious adverse events, underscore the potential of this technology. However, the modest improvements in primary outcomes such as the Gross Motor Function Measure suggest that while wearable cyborg devices hold promise, further research is needed to refine these technologies and optimize their therapeutic protocols.

## Innovative brain stimulation techniques

Transcranial photobiomodulation (tPBM) and transcranial electrical stimulation techniques like transcranial direct current stimulation (tDCS) and ranscranial random noise stimulation (tRNS) are emerging as non-invasive methods with potential in neuro-functional recovery. The study from Fradkin et al. examined the use of tPBM in young children with autism spectrum disorder (ASD). This randomized, sham-controlled trial showed better improvement of Childhood Autism Rating Scales, and it provided preliminary evidence that tPBM might reduce ASD symptoms and influence brain electrophysiology. The promising results suggested that tPBM could be a safe and effective treatment, warranting more extensive future research. In the realm of developmental dyslexia, Battisti et al. outlined a study protocol comparing the effects of tDCS and high-frequency tRNS on reading performance. This innovative study aimed to determine which stimulation technique offers the most benefit in improving reading skills in children and adolescents with dyslexia, potentially being game-changers in neurorehabilitation for developmental disorders.

## Enhancing motor recovery through electrical stimulation

Electrical stimulation continues to be a cornerstone of neurorehabilitation. Halawani et al. presented a systematic review and meta-analysis assessing contralaterally controlled functional electrical stimulation (CCFES) vs. conventional neuromuscular electrical stimulation (NMES) for stroke recovery. The analysis including 16 randomized control trials found that CCFES, particularly when using electromyographic bridges, significantly improves upper extremity function in stroke patients compared to conventional NMES. This suggests that CCFES could better harness neuroplasticity, potentially leading to more effective rehabilitation outcomes. The feasibility trial by Fujimoto et al. on combining repetitive facilitative exercise (RFE) with task-oriented training (TOT) under continuous neuromuscular electrical stimulation represents another innovative approach to stroke rehabilitation. The results of before-and-after pilot study indicated that this combined therapy is not only safe and well-tolerated but also beneficial, with patients showing clinically significant improvements in motor function and quality of movement. This combination therapy may set a new standard in neurorehabilitation, emphasizing the importance of integrating different therapeutic modalities to maximize patient outcomes.

## The role of rTMS in stroke recovery

Finally, Li et al. designed a meta-analysis on repetitive transcranial magnetic stimulation (rTMS), and provided a nuanced understanding of its efficacy in stroke rehabilitation. The study highlighted that effectiveness of rTMS was highly dependent on parameters such as the severity of hemiplegia and the stage of stroke recovery. For example, inhibitory rTMS was found to be most effective in the acute and subacute phases for less severe hemiplegia, while excitatory rTMS showed promise across various stages. These findings underscore the importance of personalized treatment plans in maximizing the therapeutic benefits of rTMS.

## A call for personalized neurorehabilitation

The seven studies reviewed here collectively emphasize the complexity of neuro-functional regeneration. While advancements in devices like HAL and interventions such as tPBM, tDCS, and rTMS offer new hope, their success hinges on a personalized approach that considers individual patient characteristics, the timing of interventions, and the combination of different therapeutic modalities. As research continues to evolve, the goal remains clear: to develop tailored rehabilitation strategies those maximize recovery and improve the quality of life for individuals with neurological disorders.

